# Differential gene expression during the moult cycle of Antarctic krill (*Euphausia superba*)

**DOI:** 10.1186/1471-2164-11-582

**Published:** 2010-10-19

**Authors:** Paul J Seear, Geraint A Tarling, Gavin Burns, William P Goodall-Copestake, Edward Gaten, Özge Özkaya, Ezio Rosato

**Affiliations:** 1 British Antarctic Survey, High Cross, Madingley Road, Cambridge, CB3 0ET, UK; 2 Department of Biology, University of Leicester, University Road, LE1 7RH, UK; 3 Department of Genetics, University of Leicester, University Road, LE1 7RH, UK

## Abstract

**Background:**

All crustaceans periodically moult to renew their exoskeleton. In krill this involves partial digestion and resorption of the old exoskeleton and synthesis of new cuticle. Molecular events that underlie the moult cycle are poorly understood in calcifying crustaceans and even less so in non-calcifying organisms such as krill. To address this we constructed an Antarctic krill cDNA microarray in order to generate gene expression profiles across the moult cycle and identify possible activation pathways.

**Results:**

A total of 26 different cuticle genes were identified that showed differential gene expression across the moult cycle. Almost all cuticle genes were up regulated during premoult and down regulated during late intermoult. There were a number of transcripts with significant sequence homology to genes potentially involved in the synthesis, breakdown and resorption of chitin. During early premoult glutamine synthetase, a gene involved in generating an amino acid used in the synthesis of glucosamine, a constituent of chitin, was up regulated more than twofold. Mannosyltransferase 1, a member of the glycosyltransferase family of enzymes that includes chitin synthase was also up regulated during early premoult. Transcripts homologous to a β-N-acetylglucosaminidase (β-NAGase) precursor were expressed at a higher level during late intermoult (prior to apolysis) than during premoult. This observation coincided with the up regulation during late intermoult, of a coatomer subunit epsilon involved in the production of vesicles that maybe used to transport the β-NAGase precursors into the exuvial cleft. Trypsin, known to activate the β-NAGase precursor, was up regulated more than fourfold during premoult. The up regulation of a predicted oligopeptide transporter during premoult may allow the transport of chitin breakdown products across the newly synthesised epi- and exocuticle layers.

**Conclusion:**

We have identified many genes differentially expressed across the moult cycle of krill that correspond with known phenotypic structural changes. This study has provided a better understanding of the processes involved in krill moulting and how they may be controlled at the gene expression level.

## Background

### Importance of krill

The pelagic crustacean order Euphausiacea has a worldwide distribution, consisting of 86 species that range from a few millimetres to 15 cm in body length. Antarctic krill (*Euphausia superba*; hereafter referred to as krill) is one of the largest euphausiid species (up to 6.5 cm from the front of the eye to tip of the tail) and is found exclusively in the Southern Ocean. Krill are the most prominent euphausiid species in terms of biomass, with estimates in the range of 100 to 500 million tonnes (wet mass) [1]. They are an important resource for large numbers of higher predators as well as for commercial fishing. There is presently much interest in obtaining a greater understanding of their physiology and life-cycle to assist with their effective management as a resource [2].

### Crustacean moulting

Moulting is an important and on-going process of physiological change in the life-history of all crustaceans. To moult, individuals must loosen the connectives between their living tissues and the extracellular cuticle, escape from the confines of this cuticle relatively rapidly, take up water, expand the new flexible exoskeleton and then quickly harden it for defence and locomotion. The act of ecdysis itself may only take a few moments (from seconds to minutes) but the processes building up to that point take much longer, with dramatic changes in physiology and biochemistry taking place over the course of the moult cycle. In particular, meticulous control of regulatory proteins and genes is required to form and harden a new exoskeleton as well as to dissolve, reabsorb and shed the old one [3-5]

### Krill moulting

The process of moulting is particularly prominent in euphausiids since it continues throughout adult life at a relatively high frequency [6]. Krill have the capacity to shrink or grow at each moult [7] and frequent moulting could allow individuals to adjust body size according to prevailing conditions [8]. Regular renewal may also decrease the period over which external parasites can settle and penetrate the relatively thin cuticle [9]. The high frequency and cost, in terms of energy and vulnerability, of moulting in euphausiids, implies that this process is under strong selective pressure.

### Krill cuticle and moult cycle

The organic matrix of the crustacean cuticle is a complex structure composed mainly of α-chitin microfibrils embedded in a protein matrix. These are mainly stacked in chitin/protein lamellae, called laminae. Morphologically, there are three different layers in the cuticle of krill: an outermost epicuticle followed by an exocuticle and an endocuticle [10]. The epicuticle is the only layer to contain glycoproteins in addition to the chitin/protein matrix but makes up only 1.6% of total cuticular thickness. The exocuticle makes up 20% of the cuticle. Both the epi- and exocuticle are deposited before the krill sheds its old shell in the moult. The remainder of the cuticle is endocuticle, which is quickly built up in the postmoult period. The epidermis lies beneath these layers and is responsible for forming the new cuticle and secreting enzymes that digest the old one. Cell processes of the epidermis travel through pore canals in the new cuticle to reabsorb digested old cuticle components [10].

Drach [11] described distinct morphological stages within the crustacean moult-cycle. This staging system was adapted by Buchholz [12,13] for euphausiids and is summarised below:

• Postmoult (Stages A and B) - a soft cuticle

• Intermoult (Stage C) - a consolidated and hardened cuticle

• Early Premoult (Stages D0 to D2) - exuvial cleft between the old cuticle and the epidermis, followed by the progressive development of an underlying new cuticle

• Late premoult (Stage D3) - old cuticle is soft

• Ecdysis - the old cuticle detaches from the body

Buchholz and Buchholz [14] carried out an ultrastructural examination of the moult-cycle stages of krill and revealed processes that underlie these morphologically distinct stages. For instance, the number of laminae was found to vary considerably over the moult cycle, with the maximal thickness of the endocuticle occurring during the intermoult phases, where maximal consolidation and hardness of the cuticle is observed. Laminae in the old cuticle were found to reduce dramatically only in late premoult, when the krill cuticle becomes soft. Tonofibrillae (or intercuticular fibres) remain connected to the old cuticle until late premoult, allowing individuals to remain active throughout preparations for moult. Epidermal cells were largest around the time of moult, reflecting an uptake of water to increase body size before the hardening of the new cuticle.

Distinct moult-related phases have also been recognised at the biochemical level [15,16]. Enzymes that digest the old cuticle such as β-N-acetylglucosaminidase (β-NAGase) and chitinase reach peak expression in premoult [15]. It is believed that such enzymes are under the control of ecdysteroids, which also show peaks in activity during the premoult phase. This activity is accompanied by an increase in the haemolymph of glucosamine, a break-down product of chitin. Such products are likely to be channelled into the metabolic pool and be reutilised in the reconstruction of the new cuticle. Such resorption and reutilisation of cuticular components is a key process in euphausiids not common to many other crustaceans.

### Conflict between cuticle formation and resorption

When the epidermis is secreting new cuticle, crustaceans must also digest and reabsorb the inner layers of the old cuticle without the new cuticle being digested [17]. This demands a tightly controlled sequence of events with adequate separation of counteracting enzymes. A number of mechanisms may facilitate this complex procedure. For instance, digestive enzymes may be secreted in inactive forms with exposure to accompanying activator molecules only occurring at precise times and locations. Another mechanism is to enclose enzymes within coated vesicles that only release their contents within appropriate environments. Physical separation of enzymes through cuticular barriers may also take place when inner and outer cuticles are present. The extent to which krill use these mechanisms in coordinating their moult cycle requires further investigation.

### Aims of the study

The molecular events associated with cuticle formation remain poorly understood in crustaceans [4,5,18]. Our aim was to identify genes involved or associated with the formation and recycling of cuticle in krill and study their expression patterns throughout the moult cycle. We used cDNA microarray technology to achieve this aim, representing the first time such an approach has been applied to any euphausiid species. Combining this with detailed moult staging allows us to obtain one of the most highly resolved molecular insights into moult-cycle processes yet achieved in any crustacean species.

## Results and Discussion

### Experimental design

In our experiment, biological replicates from each of the moult stages, *A/B*, *C*, *C late*, *D*_*0*_, *D*_*1*_, *D*_*2*_, and *D*_*3 *_were competitively hybridised against a control group consisting of a pool of *C early *krill. *C early *was considered the most suitable control since it is one of the easiest stages to categorise (meaning that the chance bias created by misidentification was relatively small) and also it is the stage where comparatively few processes related to the krill moult cycle appear to be underway [14]. There is no 'rest' stage in the krill moult cycle [15] and *C early *is when the last laminar layers are added to achieve full consolidation and hardening of the fresh cuticle. Therefore, it must be taken into account that certain processes related to cuticle synthesis are within the control gene expression pool such that stages where there is no cuticle synthesis will appear as being down regulated.

Our design compared all other moult stages with a single reference time-point (*C early*). This particular design has the advantage of being able the best to assess the relative changes in gene expression between the different moult stages and *C early *[19]. However, it does also mean that the gene expression features unique to this control stage are unresolved. There are other potential ways in which experiments of this sort can be designed. Kuballa et al [18] and Kuballa and Elizur [20] designed a microarray experiment in which gene expression in consecutive moult stages were compared. This has the advantage of resolving subtle variations in gene expression from one moult stage to another [19]. However, an assessment of gene expression level over the entire moult cycle is difficult since there is no single reference point. It also relies on none of the moult stages being misidentified, which can be an issue in smaller crustacean species such as krill, where moult stage phenotypic differences may be subtle. Another approach is to generate a reference that is a combination of all moult stages. This also allows each moult stage to be resolved. However, it does bias the results towards those genes where there are many fold differences in expression over the moult cycle in order to be distinguishable above the expression levels already in the control. Genes where expression is temporally limited and/or small in amplitude have a higher chance of being unresolved through this approach. We therefore considered the single time-point reference approach most appropriate for the purposes of our study aims.

### Microarray analysis

**Figure 1:**
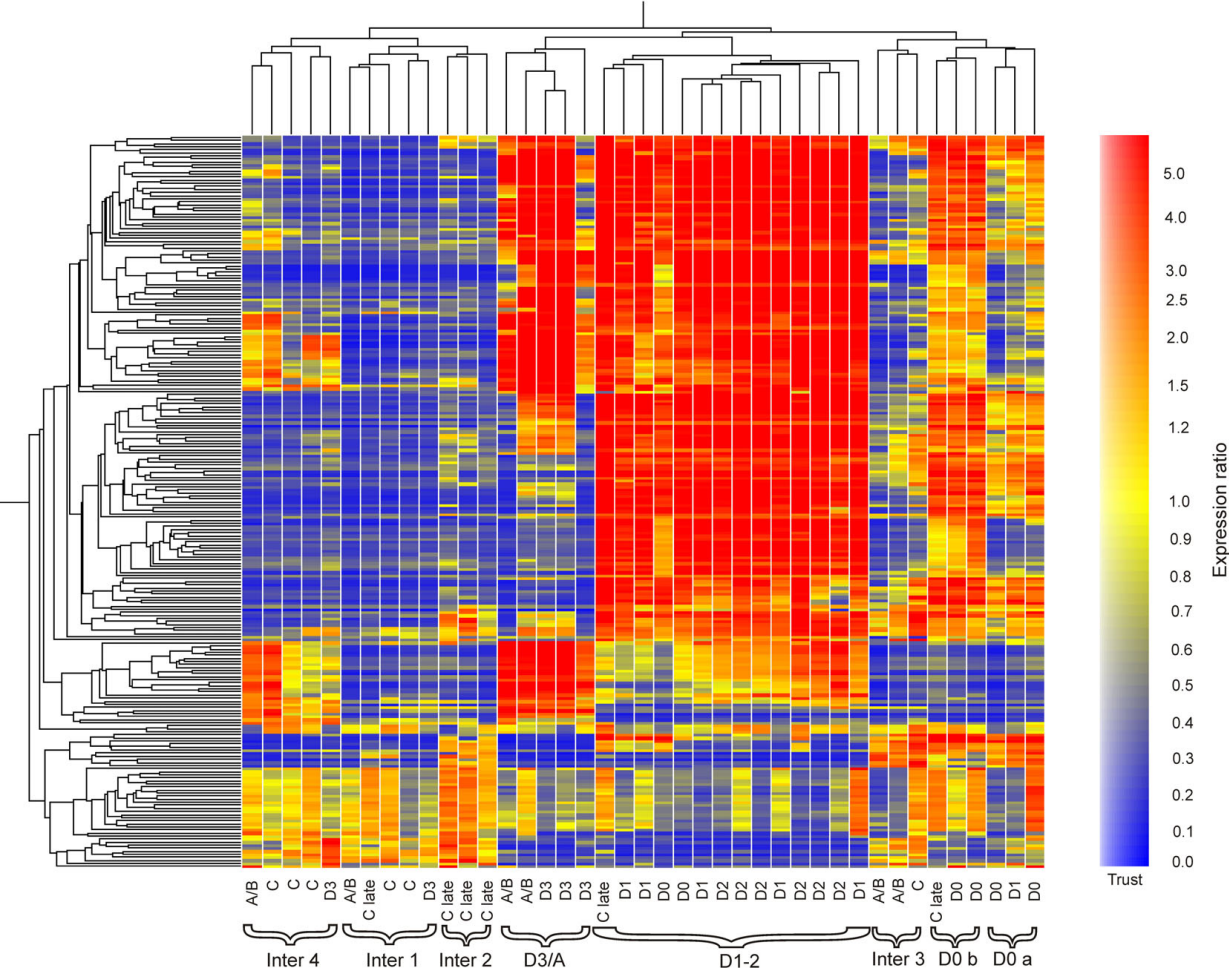
**Condition tree of transcripts significantly differentially expressed more than twofold between moult stages (similarity measure: Pearson correlation; clustering algorithm: average linkage)**. Expression colour bar denotes ratio relative to control.

**Figure 2:**
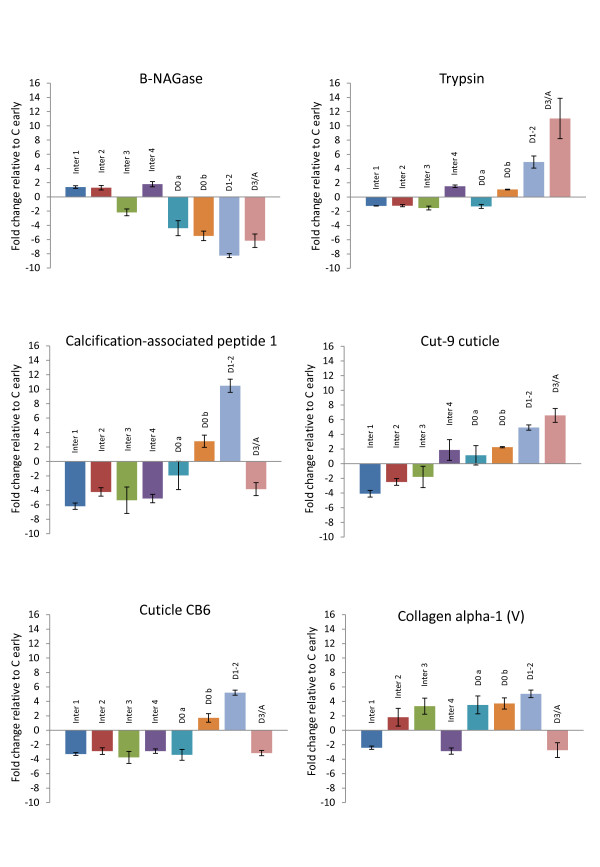
**Expression profiles of genes across the moult cycle relative to the moult cluster groups.** Fold change is indicated on the vertical axis. Error bars indicate the standard error of the mean. Quantitative PCR analysis of five of these genes indicated general agreement with the expression patterns shown here (see *additional file *2 for details).

**Figure 3:**
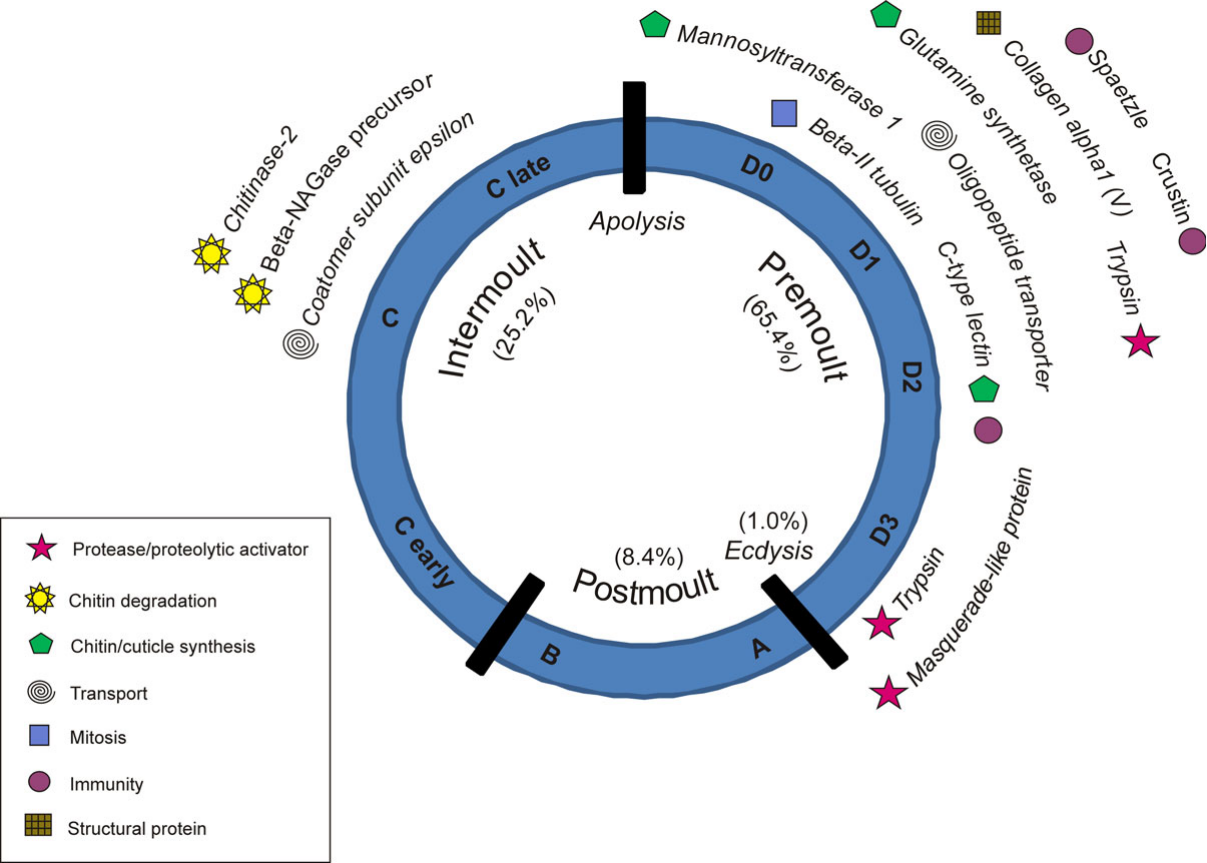
**The moult cycle of *Euphausia superba *showing the moult stage (stages A to D3) and moult phase (pre-, inter- and post-moult)**. The proportional length of time spent in each phase of the moult cycle is shown in brackets. A selection of genes with proposed functions is shown adjacent to the moult stage where they were up regulated more than two fold relative to the *C early *control.

The one-way ANOVA identified 1,110 transcripts that showed significant differential expression between moult stages (false discovery rate adjusted p < 0.05), although this does not include C *early *because of its use as a control. Of these, 239 were differentially expressed more than twofold relative to the *C early *control. A condition tree of these differentially expressed transcripts (Figure 1) shows eight cluster groups that were each dominated by a particular moult stage. The respective dominant moult stages were reflected in the designated names of these eight groups, which, in order of the moult-cycle sequence, were: Inter1, Inter2, Inter3, Inter4, D0a, D0b, D1-2, D3/A. It is to be noted that Inter1 to 4 represent different phases of expression during the intermoult period but their sequential order is uncertain. For each cluster group, transcripts that were differentially expressed more than twofold relative to *C early *are listed with fold changes, top BLASTX and tBLASTX hits (E <1e^-5^), Gene Ontology identifiers and significant (E <1e^-5^) Pfam protein domain matches in *additional file *1. Expression profiles of selected transcripts relative to the cluster-groups, ordered according to the moult-cycle sequence, are shown in graphical format in Figure 2. The expression profiles of five of these transcripts were validated by qPCR (see *additional file *2). From this type of analysis, sequence matching to NCBI databases using BLASTX and tBLASTX, different classes of transcripts were identified with potentially different functions. The sequence of up regulation of these genes relative to the moult cycle is illustrated in Figure 3.

### Cuticle genes

Many transcripts that were differentially expressed more than twofold relative to the *C early *control had significant sequence homology to arthropod cuticle genes. A number of these transcripts showed large changes in expression over the moult cycle. For instance, one transcript (Es_0600_05A06), a homologue of the calcified cuticle protein CP14.1 [GenBank: ABB91676], was down-regulated 11 fold during the intermoult stage Inter1 and up regulated by the same fold change in stage D1-2. A total of 26 different cuticle genes were revealed (Table 1), with each corresponding protein containing one of three different domain types: cuticle_1, chitin_bind_4 and CBM_14 - also known as the Peritrophin-A domain (found in chitin binding proteins [21]), as identified through NCBI and Pfam database searches. Table 1 illustrates that, during intermoult, almost all of the cuticle genes were down regulated, while in early premoult (stage D1-2), the reverse occurred. During D3/A, the number of cuticle genes up regulated compared to D1-2 was lower, with approximately half of the differentially expressed genes in this moult stage down regulated. This can be seen in graphical format in Figure 2 where transcripts homologous to the cuticle genes, calcification-associated peptide 1 and cuticle CB6 were down regulated in D3/A while the Cut-9 cuticle transcript was up regulated during this moult stage. All the down regulated genes in D3/A were of the chitin_bind_4 type.

**Table 1:** Expression profiles of cuticle genes identified through BLASTX analysis differentially expressed more than twofold relative to *C early *(p < 0.05).

Cuticle gene BLASTX hit	Domain	Int1	Int2	Int3	Int4	D0a	D0b	D1-2	D3/A
ABB91676.1|calcified cuticle protein CP14.1 [Callinectes sapidus]	Chitin_bind_4	↓	↓		↓	↑	↑	↑	↑
ABU41097.1|putative cuticle protein [Lepeophtheirus salmonis]	Chitin_bind_4	↓			↓		↑	↑	
AAV28476.1|arthrodial cuticle protein AMP8.1 [Callinectes sapidus]	Chitin_bind_4	↓	↓	↓			↑	↑	↑
AAV28479.1|calcified cuticle protein CP8.2 [Callinectes sapidus]	Chitin_bind_4	↓			↓	↑	↑	↑	↓
ABM54465.1|cuticle protein CB6 [Portunus pelagicus]	Chitin_bind_4	↓	↓	↓	↓	↓		↑	↓
BAC81566.1|calcification-associated peptide-1 [Procambarus clarkii]	Chitin_bind_4	↓	↓	↓	↓			↑	↓
BAD16776.1|calcification-associated peptide-2 [Procambarus clarkii]	Chitin_bind_4	↓		↓	↓			↑	↑
ABC26005.1|arthrodial cuticle protein AMP16.3 [Callinectes sapidus]	Chitin_bind_4	↓			↓	↑	↑	↑	
ABB91677.1|arthrodial cuticle protein AMP16.5 [Callinectes sapidus]	Chitin_bind_4	↓	↓	↓	↓	↑	↑	↑	
ABR27687.1|cuticle proprotein proCP5.2 [Callinectes sapidus]	Cuticle_1	↓	↓	↓			↑	↑	↑
ACO12089.1|Cuticle protein 19 [Lepeophtheirus salmonis]	Chitin_bind_4	↓	↓	↓	↓			↑	↓
BAA90876.1|DD9B [Marsupenaeus japonicus]	Chitin_bind_4	↓	↓	↓	↓		↑	↑	↑
FAA00634 | TPA: putative cuticle protein [Bombyx mori]	Chitin_bind_4	↓			↓		↑	↑	
P81389.1| Cuticle protein AMP5 [Homarus americanus]	Chitin_bind_4						↑	↑	↑
P81575.1| Cuticle protein AM/CP1114 [Cancer pagrus]	Chitin_bind_4	↓					↑	↑	↑
XP_001868236.1|cuticle protein [Culex quinquefasciatus]	Chitin_bind_4	↓	↓		↓		↑	↑	↓
XM_969006.2|PREDICTED: similar to Cuticular protein 47Ef CG13214-PA [Tribolium castaneum]	Chitin_bind_4	↓			↓			↑	
BAF73806.1|calcification associated soluble matrix protein 2 [Procambarus clarkii]	Chitin_bind_4	↓	↓	↓	↓	↑	↑	↑	↓
ABM54460.1|cuticle protein CUT9 [Portunus pelagicus]	Cuticle_1	↓	↓				↑	↑	↑
ABN13583.1|putative cuticle protein [Artemia franciscana]	Chitin_bind_4					↓			↓
NP_001161908.1|cuticular protein analogous to peritrophins 3-D1 [Tribolium castaneum]	CBM_14	↓	↓		↓	↑	↑	↑	↑
EF102012.1|Portunus pelagicus cuticle protein CB7-like mRNA	Chitin_bind_4						↑	↑	
XP_001961118.1|GF13710 [Drosophila ananassae]	Chitin_bind_4				↓				
EF101994 | Portunus pelagicus cuticle protein CUT2 mRNA	Cuticle_1	↓	↓				↑	↑	↑
P82119 | RecName: Full = Cuticle protein 6 [Blaberus craniifer]	Chitin_bind_4	↓	↓				↓		↑
ACL79887 | cuticle protein [Rimicaris exoculata]	Cuticle_1						↓		

Krill are almost unique among crustaceans in that they do not have a calcified exoskeleton [10] and this is supported by the types of cuticle genes found differentially expressed in this study. The proteins of most of the differentially expressed cuticle genes have either been isolated from uncalcified, arthrodial membranes of calcifying crustaceans, such as AMP8.1, AMP16.3 and AMP16.5 from *Callinectes sapidus *[22,23], or found to have an inhibitory role in calcification, for example the calcification associated peptides 1 and 2, isolated from the crayfish *Procambarus clarkii *[24,25]. There were only four cuticle proteins that contained the cuticle_1 domain, and although proteins with this domain have been isolated from the calcified cuticle of the crab, *Cancer pagurus *[26] and lobster, *Homarus americanus *[27], they did not include these four. The remaining cuticle genes have either yet to be fully annotated, such as cuticle protein CB6 from *Portunus pelagicus *[18] or have been described from non-calcifying crustaceans, such as *Lepeophtheirus salmonis *or insects such as *Tribolium castaneum*.

### Sequence of expression of the cuticle genes and chitin synthesis

The first visible sign that an individual krill is about to undertake the process of moulting is apolysis (stage D0), the point at which an exuvial cleft opens between the old cuticle and the epidermis. At the level of gene expression, we found a number of genes involved with cuticle synthesis that were up regulated at this stage. For instance, there was a threefold up regulation of glutamine synthetase, which encodes for an enzyme involved in the synthesis of glutamine from glutamate and ammonia [28,29]. Glutamine, along with fructose 6-phosphate, is used in the synthesis of glucosamine [30], an amino sugar that forms long chain N-acetylglucosamine polymers that make up chitin. A transcript with significant sequence homology to mannosyltransferase 1 [GenBank: XP_0024163.86], encoding for an enzyme (EC 2.4.109) belonging to the glycosyltransferase (EC 2.4) family of enzymes [31], was also up regulated at this point in the moult cycle. The glycosyltransferases include chitin synthase (EC 2.4.1.16), an enzyme that synthesises chitin polymers through catalysing glycosidic bonds between monosaccharides [32]. Furthermore, we observed a simultaneous up regulation in C-type lectin, which may be involved in the production of cuticular glycoproteins [20].

The period between early and late premoult is when the epi- and exocuticle layers are laid down. The epicuticle layer only makes up 1.6% of the thickness of the completed cuticle, but it plays a significant role since it is the only layer to contain glycoproteins. Amongst a range of functions, glycoproteins are known to decrease water friction [33] so they may be vital in optimising swimming efficiency in these highly motile organisms. The formation of epi- and exocuticle layers already creates a barrier between the old cuticle and the rest of the organism long before the point of ecdysis.

We also observed up regulation of two transcripts homologous to beta-II tubulin [GenBank: U66319.1] during early premoult. These genes encode for a globular protein involved in the formation of microtubules, which are involved in mitosis. Early premoult is when cell division occurs in the epidermis. The epidermal layer may appear folded within the confines of the cuticle during premoult, in readiness for expansion once the old cuticle is shed [17].

BLAST analysis identified a transcript up regulated during premoult with homology to collagen alpha 1 (V) [GenBank: XP_001942985.1], a protein that controls the formation and organisation of collagen fibrils [34]. Bundles of collagen fibrils form insoluble fibres with high tensile strength that, amongst many other functions, are prominent in extracellular matrices. These matrices assist in growth and development through providing a framework on which to organise complex structures. Exoskeletal setae that are found on the numerous appendages of krill to facilitate swimming and the capture and processing of food, are notably complex in their diversity and structure. Early stages in the development of these setae are observable during the premoult stage and even serve as a diagnostic feature of this moult stage. Interestingly collagen alpha 1 (V) may also have a role in binding chitin structures since Pfam analysis of this transcript identified a CBM_14 domain found in chitin binding proteins [21].

### Resolving the conflict between cuticle formation and resorption

The fact that a barrier between the old cuticle and the rest of the organism is in place by mid-premoult places some restrictions on the krill in terms of recycling components from the old cuticle. Firstly, this barrier prevents digestive enzymes with large molecular weights from being secreted into the exuvial cleft. Secondly, breakdown products cannot be freely transferred into the metabolic pool within the haemolymph. We found a number of features in the sequence of gene expression which demonstrate how krill overcome this potential conflict.

In terms of digestive enzymes, we observed that the highest expression levels of various β-NAGases and chitinase 2 occurred during intermoult. This is in advance of apolysis and the opening of the exuvial cleft. These enzymes must therefore be secreted into the cleft before the epicuticle barrier starts to form. This agrees with Buchholz and Buchholz [14] who considered that discharge of digestive enzymes into the exuvial cleft must commence prior to stage D1 (mid premoult).

Nevertheless, peak activity in these digestive enzymes does not occur until mid to late premoult [15] meaning that there must be a lag in their activity. We found evidence of two means by which krill may engineer such a lag at the gene expression level. Firstly, during the time that digestive enzyme genes were expressed, there was a simultaneous up regulation of a gene (coatomer subunit epsilon [GenBank: ACO15331]) encoding for a protein required for budding of vesicles from Golgi membranes. Packaging the digestive enzymes within vesicles prevents them coming into contact and digesting cuticle until the vesicles have broken down. Compere et al [35] found that exolysosomes first appeared in decapods during apolysis (stage D0) and did not appear to lose their contents into the exuvial space until late premoult (stage D2).

We also observed that most of the β-NAGase related gene transcripts had significant sequence homology (E <1e^-5^) to a β-NAGase precursor [GenBank: NP_001037466] (although this was not the top BLASTX hit). This requires limited proteolysis by trypsin in order to become activated [36]. The activation may be triggered by a transcript (Es_0051_09G11) with significant sequence homology to trypsin, which is up regulated by 4.1 fold in D1-2 and 8.2 fold in D3/A (Figure 2). Therefore, the enzymes can be transported to sites of activity without harming any other structures, including any newly synthesised cuticle. The major peak of cuticle digestion occurs over a relatively short time window, in late premoult (stage D3) when the number of laminae in the old cuticle decrease rapidly and the krill becomes soft [10]. This mechanism of producing precursor enzymes allows stocks to be accumulated with only rapid proteolysis required to facilitate a relatively instantaneous increase in cuticle digestion activity.

Also during premoult (stages D1-2), we found that a transcript homologous to a predicted oligopeptide transporter [GenBank: XP_002196515] was up regulated more than two fold. Although the newly formed epi- and exocuticle layers form a barrier between the old cuticle and the rest of the organism, it is essential that there is exchange of some components if any recycling of the old cuticle is to occur. The transporter is likely to be part of an intercellular transport mechanism in which low molecular weight products are passed into and out of the exuvial space. The increasing β-NAGase activity, in concert with other enzymes such as endochitinases and proteinases, accelerates the decomposition of the old cuticle during the last stages of the moult cycle [16]. The liberated chitin oligomers and, in particular, the amino sugars are resorbed via the epidermis [14]. Fine extensions of the epidermal cells extend transversely through the new cuticle via pore canals, coming into contact with the exuvial space [14]. These pores are small enough to allow small molecules of digested cuticle (amino acids, N-acetylglucosamine, glucosamine etc.) to enter and be reabsorbed, but do not permit entry of large enzyme molecules such as the β-NAGases [17]. The same pores may also allow the passage of activator molecules such as trypsin to flow outwards into the exuvial space. These pores eventually become cut off when the new cuticle is finally complete during intermoult [10].

### Hormonal control of digestive enzymes

As mentioned earlier, we found that many of the β-NAGase-like transcripts encoded for precursors that required proteolysis by enzymes such as trypsin in order to become active and that we also found a transcript homologous to trypsin up regulated during premoult. Shechter et al [4] found that trypsin was amongst several enzymes that were differentially expressed over the moult cycle of red claw crayfish and that expression levels were responsive to levels of ecdysteroid. Ecdysteroids are known to play a coordinating role in the moult cycle of many arthropods [37,38] through acting on ecdysteroid responsive genes. In krill, Buchholz [15] reports that there are several peaks in ecdysteroid activity during the premoult phases, coinciding with peaks in chitinase and β-NAGase activity. Our results suggest that the actual mechanism of control may act through the response of trypsin to the titre of ecdysteroid, which then regulates the level of digestive enzyme activation.

### Ecdysis

We found transcripts with significant sequence homology to genes encoding proteases such as trypsin and masquerade-like protein [GenBank: ABY64694.1] were up regulated during late premoult (stage D3/A). Although we have already discussed the role of these enzymes as activators, they may also function in breaking down protein directly. During the 10 days between apolysis and ecdysis in krill, tonofibrillae (or intercuticular fibres) remain connected to the old cuticle until just before the point of ecdysis (late premoult D3-4). This is also observed in insects [39,40] and in other crustaceans [41,42]. The up regulation of the proteases may be part of the mechanism that acts to break down tonofibrillae and so release the old cuticle.

### Protective measures

During early postmoult the krill still have a soft cuticle and so may be more vulnerable to pathogenic attack. It is interesting then, that transcripts homologous to a number of immune-related genes were up regulated during D1-2. As with all crustaceans, krill do not have an adaptive immune system and instead rely upon an innate immune system composed of cellular and humoral immune responses for protection against pathogens.

Part of the humoral response involves the production of antimicrobial peptides, small cationic molecules that in shrimps consist of three main families: penaeidins, antilipopolysaccharide factors and crustins. Two transcripts homologous to the gene encoding for crustin 4 from *Panulirus japonicus *[GenBank: BAA90876.1] were up regulated more than threefold during D1-2. Activation of antimicrobial peptide genes occurs through the Toll and Imd (immune deficiency) signalling pathways in *Drosophila *[43], and so it is revealing that a transcript homologous to a gene encoding for spätzle 2-like protein [GenBank: ABU41133.1] was up regulated more than sixfold during D1-2, as spätzle is a known activator of the Toll signalling pathway [44]. Indeed, Shi et al [45] demonstrated up regulation of crustin gene expression in the Chinese shrimp, *Fenneripenaeus chinensis *following injection of recombinant spätzle protein. Spätzle itself is synthesised as an inactive proprotein and is activated by cleavage with serine proteases [46] including trypsin [44].

## Conclusion

The objective of the present study was to use a cDNA microarray that we developed for krill to document the sequence of expression of genes over the moult cycle. We found that many of the genes identified as being differentially expressed over the moult cycle have already been described in other studies, particularly those focussed on arthropod cuticle synthesis. We also found genes relating to many other physiological processes were differentially expressed according to moult cycle stage, such as protein processing and synthesis, mitosis, extracellular-matrix construction, proteolytic activation and immunity. Our study showed that gene expression related to recognisable morphological changes occurring in premoult, postmoult and intermoult takes place in advance of those stages. With such a high frequency of ecdysis, the moult cycle has a dominant influence on the behavioural and physiological ecology of krill [47]. This study makes significant progress in our understanding of how this cycle operates at the gene-expression level, particularly in terms of explaining how the structural steps undertaken at each moult stage are implemented and how they might be controlled. When combined with the use of fresh material from a wide geographic area and the accurate assessment of morphological state, analysis with a cDNA microarray constitutes a powerful means for future investigations of genes and physiological processes important to the ecology of krill.

## Methods

### Sampling

Antarctic krill were caught during the Discovery 2010 cruise JR177 in January and February 2008. Krill were taken from two sampling regions, one in the vicinity of the South Orkney Islands (60.44°S to 60.53°S and 47.97°W to 48.13°W) and the other to the northwest of South Georgia (52.74°S to 53.68°S and 38.02°W to 40.07°W). Details of capture are described in Gaten et al [48]. For the cDNA library construction, krill were caught at different times of the day (00:50, 06:00, 07:00, 14:00 and 20:30) and samples flash frozen before being stored at -80°C. These krill were all caught at 60°S, except for the 06:00 and 00:50 samples which were caught at 52°S. For the moulting experiment, a tank with approximately 115 L of flowing seawater was loaded with 400+ krill caught at 60°S. Krill were maintained under a controlled light regime that mimicked that normally experienced by swarms at 60°S. After two days in these conditions, 10-20 krill were sampled each day at 20:00 and uropod length, moult stage and sex recorded. Heads were removed and preserved in RNA*later*^® ^(Applied Biosystems) following manufacturer's instructions. The head contains the eyes and brain as well as parts of the carapace, so it was the most appropriate body section to examine with respect to identifying genes involved in both the control of the moult cycle and its implementation.

### Moult staging

Moult staging was carried out on uropod segments that were separated from the main body of fresh specimens immediately prior to preservation and kept in a controlled temperature room held at approximately 2°C. The uropod segments were examined under a high powered light microscope within 3 h of separation. Each segment was moult staged following the original categorisation of Buchholz [12] updated by Buchholz [13]. A simplified version of the Buchholz [13] scheme was implemented that distinguished a total of 8 moult stages. These stages were, in sequential order: *A/B*, *C early*, *C*, *C late*, *D0*, *D1*, *D2 *and *D3*. Within this scheme, ecdysis (the loss of the old cuticle) occurs at the transition between *D3 *and *A *and apolysis (the point at which the old cuticle separates from the epidermis and a cuticular cleft appears) between *C late *and *D0*. For comparative purposes, stage *A/B *corresponds to the postmoult stage, *C early*, *C *and *C late*, the intermoult stage, and *D0*, *D1*, *D2 *and *D3*, the premoult stage.

### Construction of cDNA libraries

A cDNA library was constructed for each of the five time points (00:50, 06:00, 07:00, 14:00 and 20:30). For each library, six flash frozen krill were randomly selected from each time point and total RNA extracted from the heads (including eyes and brain) using TRI reagent (Applied Biosystems) followed by purification with RNeasy columns (Qiagen). Concentration and purity of the total RNA was determined using a NanoDrop spectrophotometer (LabTech International). One microgram of total RNA was electrophoresed on a non-denaturing 1.5% (w/v) agarose gel to check for degradation. Equal amounts of total RNA from each of the six krill were then pooled before isolating mRNA using a MicroPoly(A) Purist™ kit (Applied Biosystems). Concentration and quality of mRNA was determined as for total RNA. This mRNA (500 ng) was used in the construction of each library in pDNR-LIB using Creator SMART cDNA library kits (Clontech) and transformed into XL-1 Blue competent cells (Agilent Technologies) following manufacturer's instructions. Recombinant colonies were selected on LB agar medium + chloramphenicol. For each library 960 individual colonies were manually picked into 96 well plates with 14% (w/v) glycerol in TB + chloramphenicol, incubated overnight at 37°C and stored at -80°C.

### Sequencing

Inserts from each cDNA clone were PCR amplified with the primers M13F: 5'-GTAAAACGACGGCCAGT-3' and M13R: 5'-AACAGCTATGACCATG-3' and sequenced using the same M13R primer used in the PCR, as described by Purać et al [49]. Sequence chromatograms were processed using Geneious 4.7 [50] that trimmed vector, low quality sequence and 3'-polyadenlylate tails and generated assemblies based on a word length of 50 and gap and mismatch errors of 5%. From a total of 4,800 sequences obtained from the five cDNA libraries, 3,092 were retained for further analysis. These sequences were assembled into 302 contigs, leaving 1,624 singletons; details of the numbers of contigs and singletons assembled from individual libraries can be found in *additional file *3. A comparison between the 3,092 sequences and Antarctic krill ESTs already in GenBank [c.f. [51,52]] identified 52% of the transcripts as novel (46% of which were singletons and 6% contigs). Following sequence matching to NCBI databases using the BLASTX tool in Blast2GO (version 2.3.6 [53]), sequences from each library were further annotated with Gene Ontology identifiers [54] (*additional files *4, 5, 6, 7 and 8). All edited sequences (≥100 bp) were submitted to dbEST and given the following accession numbers: dbEST: 68794898-68797989, Genbank: GW421184-GW424275.

### Microarray fabrication

PCR amplification of all 960 clone inserts from each library was performed using amine terminated M13 primers as described by Purać et al [49]. PCR products were electrophoresed through a 1.5% (w/v) agarose gel and those that produced more than a clear, single band were flagged as failed and were not considered in downstream analysis. PCR products were transferred to 384 well plates and spotting buffer added according to the protocol of Lyne et al [55]. The array elements were then contact spotted onto activated amine-binding slides (Codelink, Surmodics) using a Genetix Q-array II robot with a 24-pin tool. Following printing, the microarrays were incubated at room temperature in a saturated sodium chloride humid chamber overnight and post-processed according to the manufacturer's instructions. In total 10,176 features were spotted on the microarray including 4,792 clones from the five cDNA libraries spotted in duplicate and the Stratagene SpotReport Alien cDNA microarray validation system. Details of the microarray can be found at ArrayExpress (http://www.ebi.ac.uk/microarray-as/aer/login?logout=true) under accession number A-MEXP-1800.

### Experimental design

Total RNA for use in the microarray hybridisations was extracted as described for the cDNA library construction above. Double stranded cDNA probes were then prepared using 500 ng of total RNA as described in Petalidis et al [56] with the addition of 1 μl Stratagene Alien mRNA spikes prior to amplification. Microarray hybridisations were performed as described in Purać et al [49]. Six biological replicates from each of the moult stages, *A/B*, *C*, *C late*, *D*_*0*_, *D*_*1*_, and *D*_*2*_, and 5 biological replicates from *D*_*3 *_(all labelled Cy3), were competitively hybridised against the control group (labelled Cy5) consisting of a pool of 10 *C early *krill. The pool consisted of equal amounts (500 ng) of amplified cDNA from each of the *C early *krill. A dye swap was performed to check for cyanine fluor effects. All krill were female, with total body lengths of between 35 mm and 51 mm (original measurements made on uropod length and converted to total body length using a population specific calibration). A single sex was chosen to eliminate any sex-dependent expression patterns. Krill were sampled at the same time of day to control for circadian related differences. Details from the microarray experiments performed here have been submitted to ArrayExpress and assigned the accession number E-MEXP-2605. Recording of microarray experiment metadata complies with Minimum Information About a Microarray Experiment (MIAME) guidelines.

### Microarray analysis

Hybridised slides were scanned at 10 μm resolution using a GenePix 4100 microarray scanner (MDS technologies). Image analysis and local background correction of spot intensities were then performed with GenePix Pro 6.0 software (MDS technologies). Array images were only used if the following quality thresholds were passed: median signal-to-background >8; median signal-to-noise >10 and mean of median background signal <250. Anomalous spots were flagged and excluded from future analyses. GenePix results files were then imported into GeneSpring GX 7.3.1 (Agilent Technologies) for further analysis.

Normalisation was performed using a 'Per Spot and Per Chip' intensity dependent (Lowess) normalisation using software defaults (20% smoothing/cutoff 10). A one-way ANOVA incorporating a Benjamini and Hochberg [57] multiple test correction (p < 0.05) was then used to find transcripts that showed significant differences between moult stages. These significantly differentially expressed transcripts were then filtered on a greater than twofold difference in expression level. All transcripts referred to herein as being up or down regulated have passed this significance test and subsequent filtering. A condition tree was generated from these transcripts (similarity measure: Pearson correlation; clustering algorithm: average linkage) in order to group together individuals that had similar expression profiles. Clusters generated from this analysis were examined for their moult stage composition and a cluster-group name was designated based on the respective dominant moult stage. The dominance of one particular moult stage was almost always evident in each of the cluster groups.

Lists of transcripts differentially expressed more than twofold relative to the control were then generated for each cluster group. Similarity to known gene sequences was performed using the BLASTX and tBLASTX tools in Blast2GO (version 2.3.6 [53]) to search the non-redundant protein sequence and nucleotide collection databases at NCBI for significant matches with a cut off e-value of <1e^-5^. Sequences were further annotated with Gene Ontology identifiers [54] using Blast2GO. The Pfam database of protein families was searched using translated differentially expressed sequences to identify protein domains such as chitin binding domains.

### Quantitative PCR

The results from the microarray were validated by performing qPCR on five transcripts indicated to be differentially expressed between moult stages. These had significant sequence homology to β-NAGase, cuticle protein CB6, cuticle protein CUT9, trypsin and collagen α1 (V). Primers were designed around each of the five transcripts, using Primer 3 [58] to generate PCR products of between 100 and 200 bp. To normalise the data, primers were also designed against a transcript with significant homology to triosephosphate isomerase [GenBank: ADD38004] that did not significantly change in expression between all moult stages on the microarray (*additional file *9).

The same total RNA samples used for the microarray experiments were also used for qPCR. Following manufacturer's instructions, one microgram of total RNA was used to make first strand cDNA using a Quantitect Reverse Transcription kit (Qiagen) that incorporates genomic DNA removal prior to reverse transcription. The cDNA (20 μl) was diluted to 80 μl and 1 μl was used as template for qPCR. The qPCR mixture consisted of 10 μl 2 × SensiMix SYBR Low-ROX mastermix (Bioline), 600 nM of forward and reverse primers, 1 μl cDNA and MilliQ water in a total volume of 20 μl. The qPCRs were performed on a Mx3000P QPCR system (Agilent Technologies) with the following cycling conditions: 95°C for 10 min, followed by 40 cycles of 95°C for 30 sec, 60°C for 1 min and 72°C for 30 sec. A dissociation curve step was then performed to ensure that only a single product had been amplified in each reaction. Standard curves were performed for each primer pair on the same plate as the experimental samples with a dilution series of cDNA. By plotting threshold crossing cycle (*C*_*t*_) values against the log_10 _of the different dilutions, PCR efficiency was calculated as E = 10^(-1/slope) ^-1, using MxPro™ (Agilent Technologies) software. For each transcript of interest, cDNA from three individuals from each of the moult stage cluster groups were run in duplicate qPCR reactions. No template and no-reverse transcriptase controls were also performed for each primer pair and cDNA respectively. Relative mean expression ratios were statistically compared between moult stage cluster groups and *C early *following normalisation against the reference gene using the relative expression software tool REST [59]. For statistical analysis of relative mean expression ratios, REST employs randomisation tests with a pair-wise reallocation that make no assumptions about the distribution of observations in populations. The software was used to perform 2000 random allocations to determine if the results were due to chance or to moult stage, with differences considered to be significant at P < 0.05.

## Authors' contributions

PJS performed library preparation, sequencing, microarray hybridisations, qPCR, data analysis, contributed to the experimental design and drafted the manuscript. GAT performed the moult staging, analysis and interpretation of the data, contributed to the conception and design of the study and drafted the manuscript. GB printed the microarrays, supervised the microarray hybridisations and helped with the data analysis. WPGC contributed to bioinformatic analyses of the ESTs. EG performed the krill sampling and contributed to the conception and design of the study. ÖÖ contributed to construction of the microarray. ER coordinated the work and contributed to the conception and design of the study. All authors read and approved the final manuscript.

## Supplementary Material

Additional file 1 **All transcripts differentially expressed more than twofold (p < 0.05) relative to *C early *in each moult cluster group**. Each sheet in the file lists transcripts that were differentially expressed more than twofold (B&H adjusted p < 0.05) relative to *C early *for each moult cluster group. Each transcript is listed with GenBank accession number, contig/singleton identifier, top BLAST (E < 1e^-5^) description (tBLASTX hits are underlined, all others are BLASTX), E value of the BLAST hit, Gene Ontology identifiers (C = cellular component, F = molecular function, P = biological process), Pfam protein domain (E < 1e^-5^), fold change (with inverted fold change for down regulated transcripts) and false discovery rate adjusted p value. Click here for file

Additional file 2 **Quantitative PCR analysis of transcripts selected for validation of microarray data**. A table of the mean fold changes in expression of five transcripts in each moult stage cluster group relative to *C early*, as measured by qPCR. Click here for file

Additional file 3 **Details of EST clustering for each Antarctic krill cDNA library**. A table of the number of contigs and singletons generated from clustering of each individual krill cDNA library along with respective GenBank accession numbers. Click here for file

Additional file 4 **Biological process level 2 GO annotation of 0051 EST library**. A pie chart of Gene Ontology annotations at biological process level 2 of all EST sequences ≥100 bp from the 0051 cDNA library. Click here for file

Additional file 5 **Biological process level 2 GO annotation of 0600 EST library**. A pie chart of Gene Ontology annotations at biological process level 2 of all EST sequences ≥100 bp from the 0600 cDNA library. Click here for file

Additional file 6 **Biological process level 2 GO annotation of 0701 EST library**. A pie chart of Gene Ontology annotations at biological process level 2 of all EST sequences ≥100 bp from the 0701 cDNA library. Click here for file

Additional file 7 **Biological process level 2 GO annotation of 1401 EST library**. A pie chart of Gene Ontology annotations at biological process level 2 of all EST sequences ≥100 bp from the 1400 cDNA library. Click here for file

Additional file 8 **Biological process level 2 GO annotation of 2001 EST library**. A pie chart of Gene Ontology annotations at biological process level 2 of all EST sequences ≥100 bp from the 2001cDNA library. Click here for file

Additional file 9 **Primer sequences used for quantitative PCR**. A table of primer sequences used in the qPCR validation of five transcripts. Click here for file
