# Supplementary material for: Differential gene expression during the moult cycle of Antarctic krill (Euphausia superba)
Source: BMC Genomics. 2010 Oct 19;11:582. doi: 10.1186/1471-2164-11-582 (PMC3091729; doi:10.1186/1471-2164-11-582)
Supplement: Additional file 2 — Quantitative PCR analysis of transcripts selected for validation of microarray data. A table of the mean fold changes in expression of five transcripts in each moult stage cluster group relative to C early, as measured by qPCR. [file 1471-2164-11-582-S2.PDF]

Gene name/ moult stage	Mean fold change relative to *C early* by qPCR	Standard error	P value
**ß-NAGase**			
Inter 1	1.4	0.7	0.204
Inter 2	1.5	0.99	0.486
Inter 3	-1.3	3.0	0.539
Inter 4	4.2	2.6	0.001
D0 a	-2.9	4.3	0.162
D0 b	-5.0	11.6	0.087
D1-2	-24.7	39.2	0.046
D3/A	-10.0	21.1	0.024
**Trypsin**			
Inter 1	-4.4	3.3	0.338
Inter 2	-3.6	2.6	0.326
Inter 3	-11.9	8.3	0.197
Inter 4	1.7	2.3	0.730
D0 a	-2.6	1.8	0.348
D0 b	1.7	2.2	0.763
D1-2	24.4	33.5	0.023
D3/A	241.8	318.5	0.053
**Cut9 cuticle**			
Inter 1	-11.4	8.2	0.037
Inter 2	-4.9	3.5	0.373
Inter 3	-1.9	1.4	0.758
Inter 4	-1.7	1.24	0.621
D0 a	1.2	1.7	0.815
D0 b	5.8	7.9	0.312
D1-2	19.4	26.5	0.001
D3/A	65.8	90.0	0.030
**Cuticle CB6**			
Inter 1	1.5	1.9	0.585
Inter 2	8.2	11.4	0.202
Inter 3	66.0	88.0	0.001
Inter 4	-1.4	1.2	0.93
D0 a	305.3	429.9	0.001
D0 b	523.2	694.4	0.001
D1-2	460.3	610.0	0.001
D3/A	1.2	1.6	0.814
**Collagen α1 (V)**			
Inter 1	1.2	1.6	0.886
Inter 2	12.7	15.9	0.099
Inter 3	33.1	43.6	0.001
Inter 4	2.0	2.5	0.533
D0 a	42.6	55.8	0.001
D0 b	59.9	71.2	0.043
D1-2	158.7	188.7	0.001
D3/A	3.5	4.2	0.321
