# Supplementary material for: Differential gene expression during the moult cycle of Antarctic krill (Euphausia superba)
Source: BMC Genomics. 2010 Oct 19;11:582. doi: 10.1186/1471-2164-11-582 (PMC3091729; doi:10.1186/1471-2164-11-582)
Supplement: Additional file 3 — Details of EST clustering for each Antarctic krill cDNA library. A table of the number of contigs and singletons generated from clustering of each individual krill cDNA library along with respective GenBank accession numbers. [file 1471-2164-11-582-S3.PDF]

| Library | Total sequences | Analysed sequences | GenBank accessions | Number contigs | Number singletons |
|---|---|---|---|---|---|
| 0051 | 960 | 640 | GW421184- GW421823 | 38 | 457 |
| 0600 | 960 | 617 | GW421824- GW422440 | 45 | 451 |
| 0701 | 960 | 640 | GW422441- GW423080 | 57 | 355 |
| 1401 | 960 | 654 | GW423081- GW423734 | 56 | 433 |
| 2001 | 960 | 541 | GW423735- GW424275 | 33 | 375 |
