# Supplementary figures and images for: Differential gene expression during the moult cycle of Antarctic krill (Euphausia superba)
Source: BMC Genomics. 2010 Oct 19;11:582. doi: 10.1186/1471-2164-11-582 (PMC3091729; doi:10.1186/1471-2164-11-582)

## Biological process level 2 GO annotation of 0051 EST library

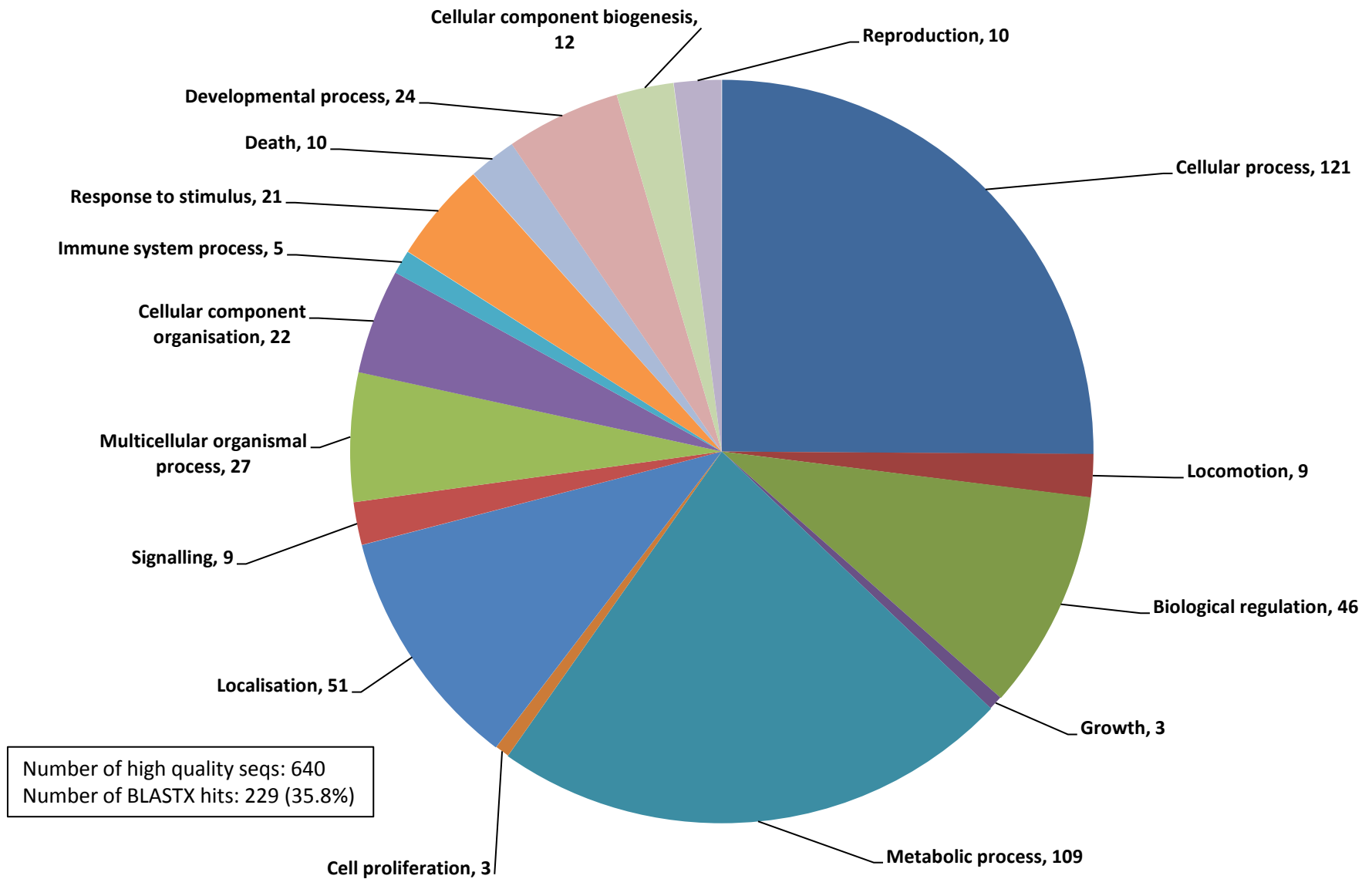

Supplement: Additional file 4 — Biological process level 2 GO annotation of 0051 EST library. A pie chart of Gene Ontology annotations at biological process level 2 of all EST sequences ≥100 bp from the 0051 cDNA library. [file 1471-2164-11-582-S4.PDF]

# Biological process level 2 GO annotation of 0600 EST library

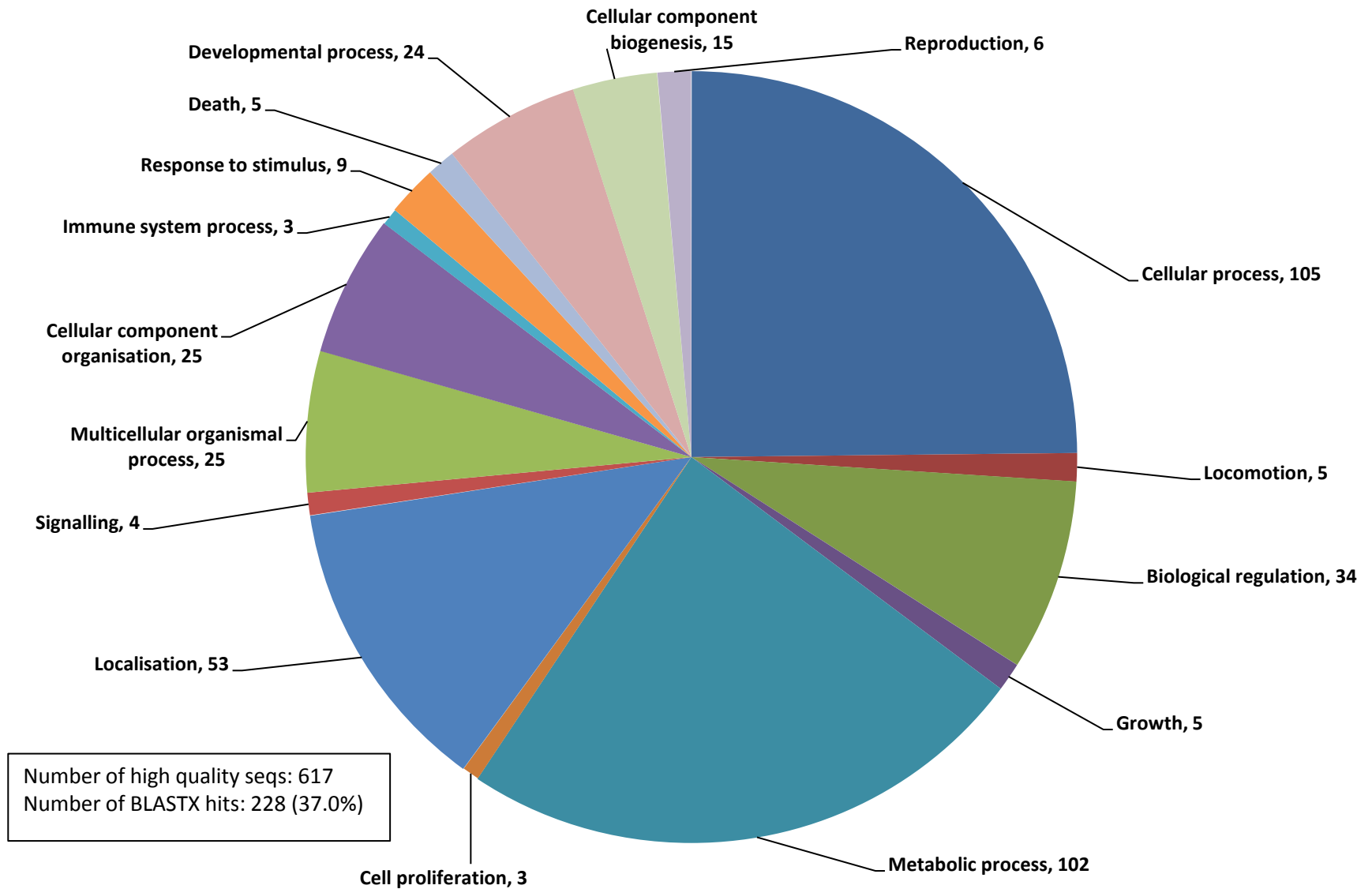

Supplement: Additional file 5 — Biological process level 2 GO annotation of 0600 EST library. A pie chart of Gene Ontology annotations at biological process level 2 of all EST sequences ≥100 bp from the 0600 cDNA library. [file 1471-2164-11-582-S5.PDF]

# Biological process level 2 GO annotation of 0701 EST library

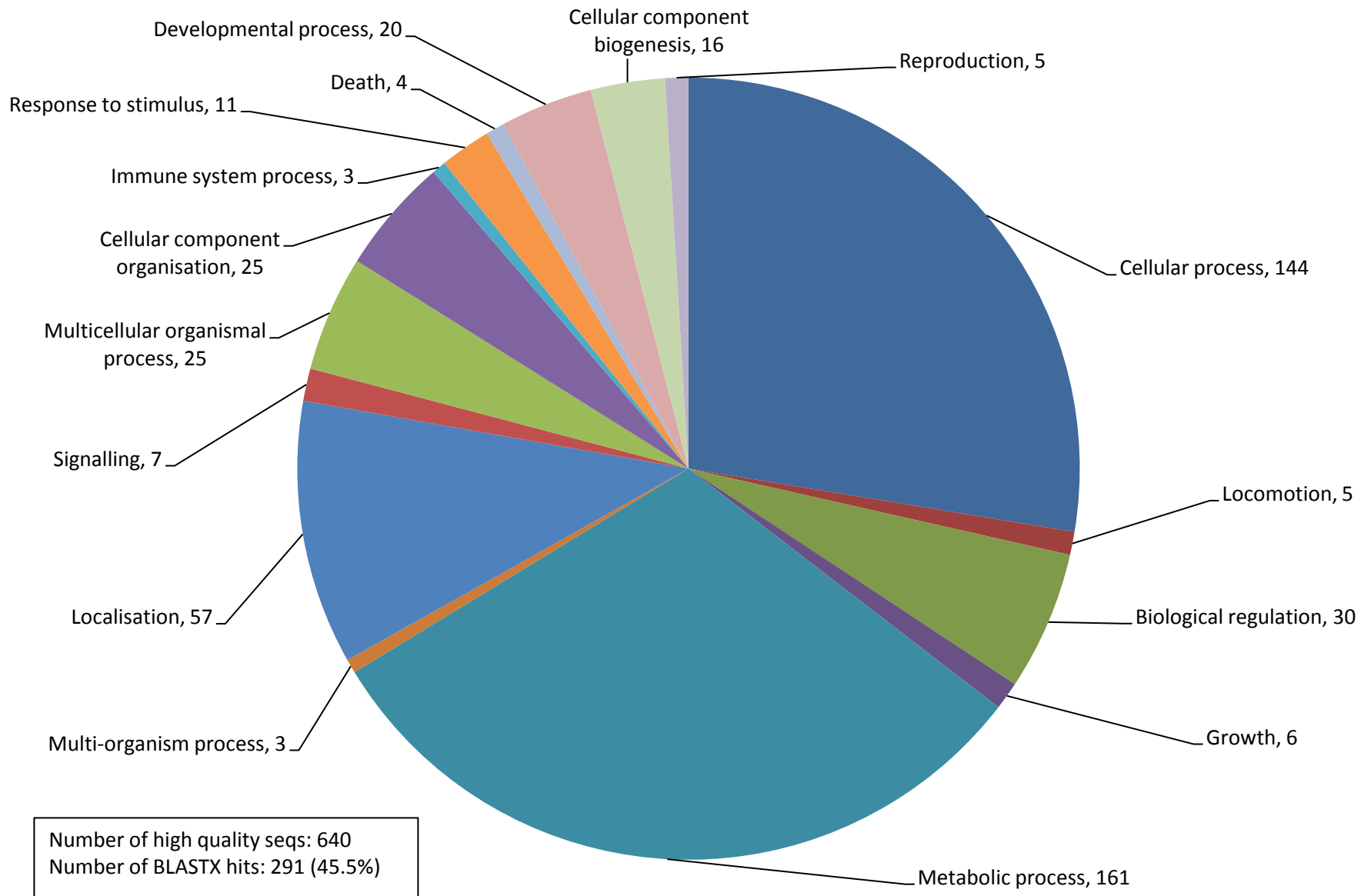

Supplement: Additional file 6 — Biological process level 2 GO annotation of 0701 EST library. A pie chart of Gene Ontology annotations at biological process level 2 of all EST sequences ≥100 bp from the 0701 cDNA library. [file 1471-2164-11-582-S6.PDF]

# Biological process level 2 GO annotation of 1401 EST library

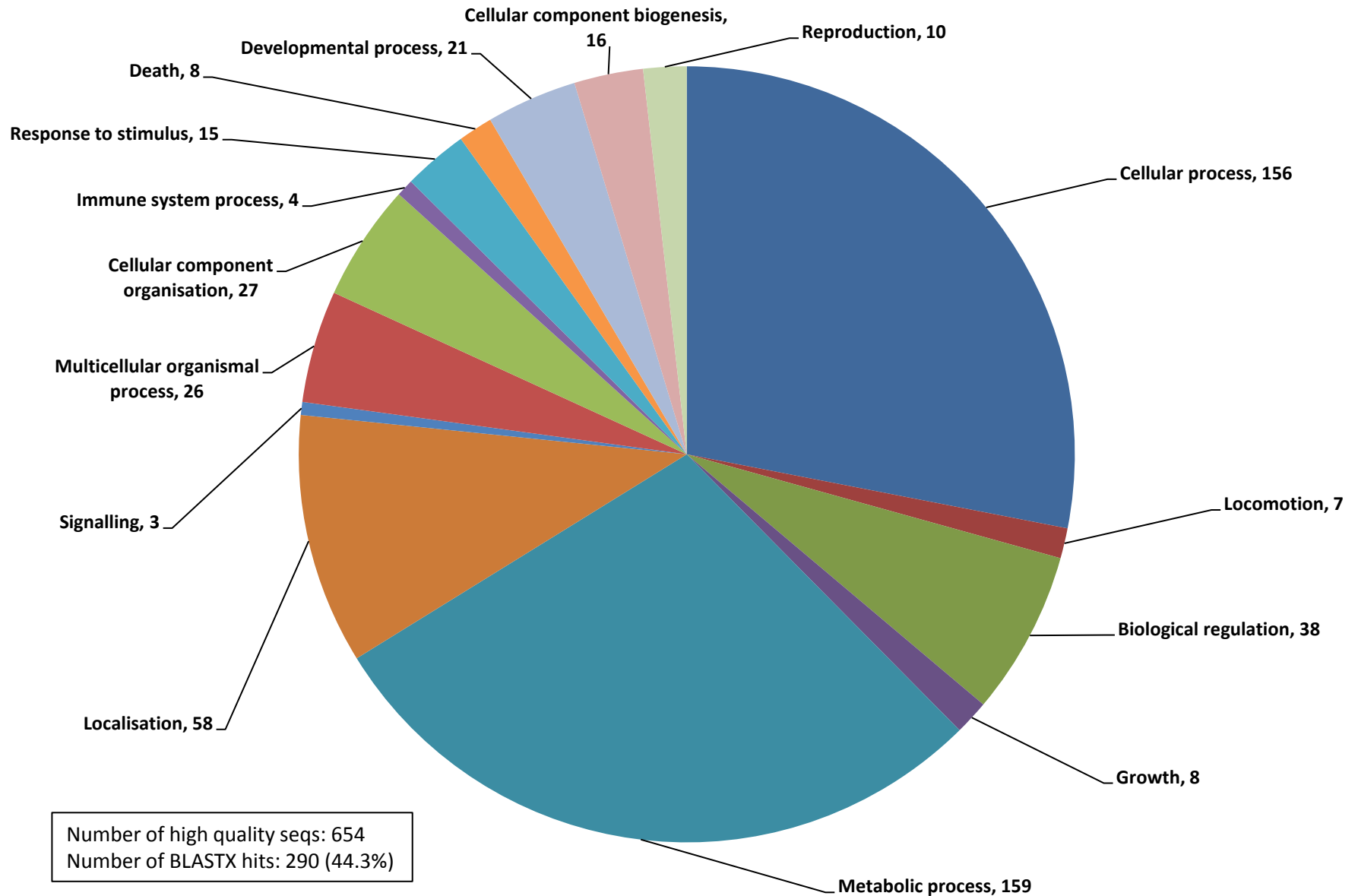

Supplement: Additional file 7 — Biological process level 2 GO annotation of 1401 EST library. A pie chart of Gene Ontology annotations at biological process level 2 of all EST sequences ≥100 bp from the 1400 cDNA library. [file 1471-2164-11-582-S7.PDF]

# Biological process level 2 GO annotation of 2001 EST library

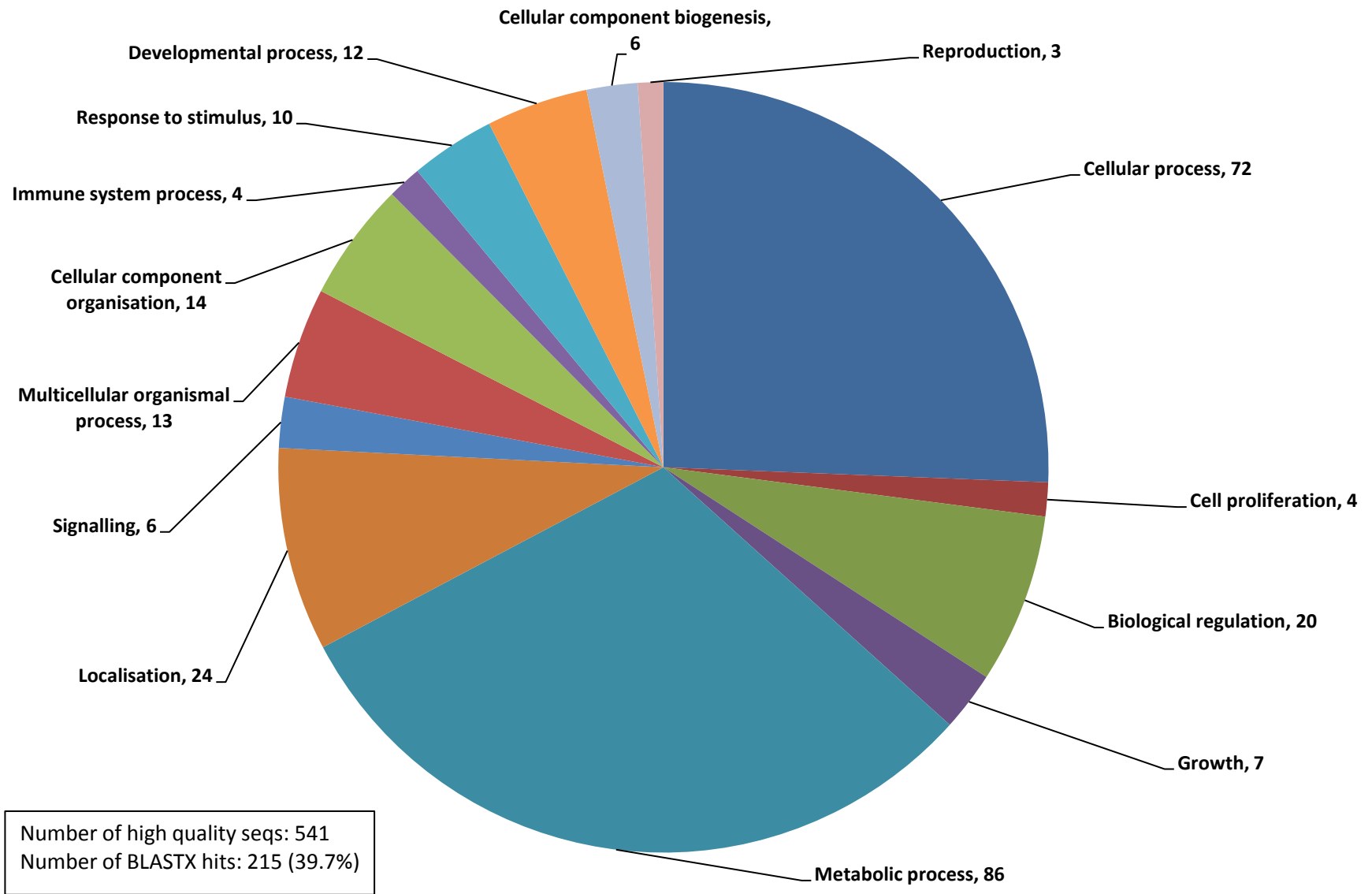

Supplement: Additional file 8 — Biological process level 2 GO annotation of 2001 EST library. A pie chart of Gene Ontology annotations at biological process level 2 of all EST sequences ≥100 bp from the 2001cDNA library. [file 1471-2164-11-582-S8.PDF]
