# Supplementary material for: Differential gene expression during the moult cycle of Antarctic krill (Euphausia superba)
Source: BMC Genomics. 2010 Oct 19;11:582. doi: 10.1186/1471-2164-11-582 (PMC3091729; doi:10.1186/1471-2164-11-582)
Supplement: Additional file 9 — Primer sequences used for quantitative PCR. A table of primer sequences used in the qPCR validation of five transcripts. [file 1471-2164-11-582-S9.PDF]

Gene name	Primer sequence 5'-3'	Product size
ß-NAGase	F: AGTGTTCTGCCGATTTTGGT<br>R: TCCTCAACAGACCCACTTCC	169bp
Cuticle CB6	F: ACCAAAGTCGTTGCCTGAGT<br>R: CGACACGACACACATCATCA	181bp
CUT9 cuticle	F: GTCAAGCGCTCACTTCCTCT<br>R: GAACAAGTCCTGAGGGACCA	115bp
Trypsin	F: GGTGCAGTTCAAAGGACCAT<br>R: ATGGCGCTCTGTTAGGAGAA	139bp
Collagen α1 (V)	F: GCCTCAGATCAATTCGGTGT<br>R: CGGTGTCTGGAACTGAGGAT	121bp
Triosephosphate isomerase	F: TTCTGTCCGCATCTTGTACG<br>R: TTCCCAGGCATTGATGATCT	139bp
